# Supplementary material for: Saccharomyces boulardii (CNCM I-745) improves intestinal damage in sepsis by remodeling intestinal flora structure
Source: Front Cell Infect Microbiol. 2025 Jul 31;15:1602792. doi: 10.3389/fcimb.2025.1602792 (PMC12350329; doi:10.3389/fcimb.2025.1602792)
Supplement: Supplementary file 1 [file DataSheet1.zip › summary/summary/8_Advanced_analyse/2_PICRUSt2_result/METACYC/Group.CW4_vs_AW4/PICRUSt2_METACYC_Group.CW4_vs_AW4_diff.pdf]

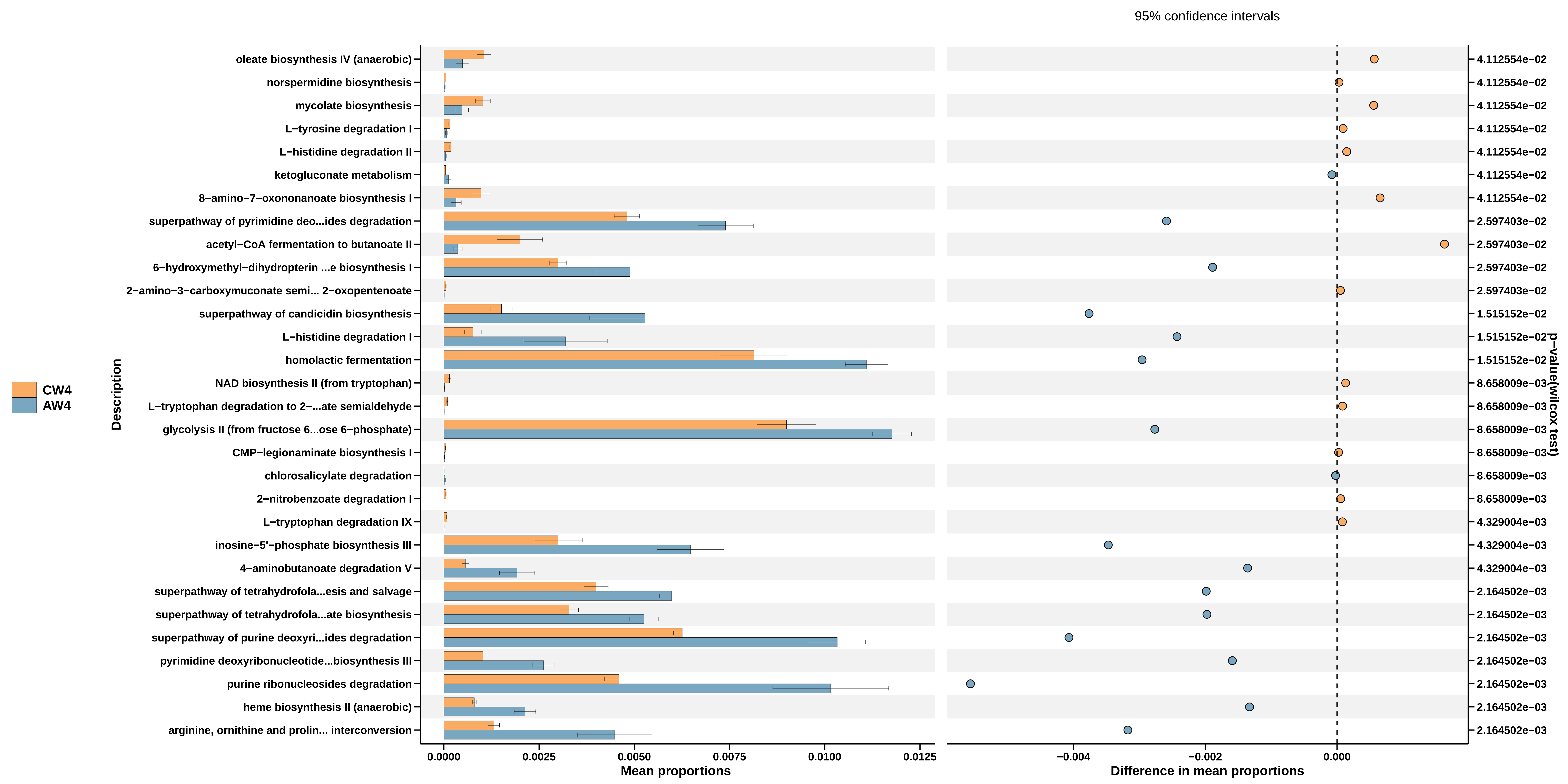

95% confidence intervals
CW4
AW4
Description
oleate biosynthesis IV (anaerobic)
norspermidine biosynthesis
mycolate biosynthesis
L−tyrosine degradation I
L−histidine degradation II
ketogluconate metabolism
8−amino−7−oxononanoate biosynthesis I
superpathway of pyrimidine deo...ides degradation
acetyl−CoA fermentation to butanoate II
6−hydroxymethyl−dihydropterin ...e biosynthesis I
2−amino−3−carboxymuconate semi... 2−oxopentenoate
superpathway of candicidin biosynthesis
L−histidine degradation I
homolactic fermentation
NAD biosynthesis II (from tryptophan)
L−tryptophan degradation to 2−...ate semialdehyde
glycolysis II (from fructose 6...ose 6−phosphate)
CMP−legionaminate biosynthesis I
chlorosalicylate degradation
2−nitrobenzoate degradation I
L−tryptophan degradation IX
inosine−5'−phosphate biosynthesis III
4−aminobutanoate degradation V
superpathway of tetrahydrofola...esis and salvage
superpathway of tetrahydrofola...ate biosynthesis
superpathway of purine deoxyri...ides degradation
pyrimidine deoxyribonucleotide...biosynthesis III
purine ribonucleosides degradation
heme biosynthesis II (anaerobic)
arginine, ornithine and prolin... interconversion
0.0000
0.0025
0.0050
0.0075
0.0100
0.0125
Mean proportions
−0.004
−0.002
0.000
Difference in mean proportions
4.112554e−02
4.112554e−02
4.112554e−02
4.112554e−02
4.112554e−02
4.112554e−02
4.112554e−02
2.597403e−02
2.597403e−02
2.597403e−02
2.597403e−02
1.515152e−02
1.515152e−02
1.515152e−02
8.658009e−03
8.658009e−03
8.658009e−03
8.658009e−03
8.658009e−03
8.658009e−03
4.329004e−03
4.329004e−03
4.329004e−03
2.164502e−03
2.164502e−03
2.164502e−03
2.164502e−03
2.164502e−03
2.164502e−03
2.164502e−03
p−value(wilcox test)
